# Integrated evidence supports a causal association between PHMG-P exposure and pneumonitis

**DOI:** 10.4178/epih.e2025073

**Published:** 2025-12-14

**Authors:** Yong Joo Park, Hye-Jin Kim, Youngmee Lee, Haewon Kim, Ha Ryong Kim, Jaiyong Kim, Hae-Kwan Cheong, Mina Ha, Kyu Hyuck Chung

**Affiliations:** 1 College of Pharmacy, Kyungsung University, Busan, Korea; 2 Humidifier Disinfectant Health Center, Environmental Health Research Department, National Institute of Environmental Research, Incheon, Korea; 3 School of Pharmacy, Korea University, Sejong, Korea; 4 Department of Big Data Research and Development, National Health Insurance Service, Wonju, Korea; 5 School of Medicine, Sungkyunkwan University, Suwon, Korea; 6 Department of Preventive Medicine, Dankook University College of Medicine, Cheonan, Korea; 7 School of Pharmacy, Sungkyunkwan University, Suwon, Korea

**Keywords:** Polyhexamethylene guanidine phosphate, Humidifier disinfectants, Chemical pneumonitis, Epidemiologic studies, Adverse outcome pathways, Causality

## Abstract

Polyhexamethylene guanidine phosphate (PHMG-P) is a guanidine-based disinfectant previously used in household humidifiers in Korea. This study evaluated whether PHMG-P exposure is causally linked to pneumonitis by integrating epidemiological, toxicological, and mechanistic evidence. We prespecified an evidence-integration framework aligned with the Office of Health Assessment and Translation/Grading of Recommendations Assessment, Development and Evaluation approaches, and applied the Organisation for Economic Cooperation and Development adverse outcome pathway (AOP) guidance to organize mechanistic evidence. We systematically identified studies and synthesized findings across 3 domains: epidemiology (age-period-cohort [APC], difference-in-differences [DID], and interrupted time-series [ITS] analyses), toxicology (animal and *in vitro* studies), and mechanism (mapping key events to an AOP). We assessed internal and external validity, coherence, and strength of evidence within and across domains. Epidemiological analyses showed that pneumonitis incidence rose during humidifier disinfectant use and declined after the 2011-2012 withdrawal, with higher risks in children and reproductive-age females. APC, DID, and ITS, including PHMG-P–specific time-series analysis, indicated increased pneumonitis incidence and mortality during exposure periods. Toxicological studies demonstrated that PHMG-P exposure resulted in epithelial injury, inflammation, fibrosis, and impaired lung function consistent with chemical pneumonitis. Mechanistic evidence linked PHMG-P exposure to epithelial damage, oxidative stress, macrophage polarization, and fibrotic changes. Multiple lines of evidence support a causal link between PHMG-P exposure and pneumonitis, underscoring the value of integrating epidemiology and toxicology to strengthen risk assessment and inform policy.

## GRAPHICAL ABSTRACT

[Fig f3-epih-47-e2025073]

## Key Message

Population-based epidemiological analyses, including age–period–cohort, difference-in-differences, and interrupted time series approaches, demonstrated increases in pneumonia diagnoses during periods of PHMG-P use and subsequent declines following its market withdrawal. Toxicological evidence, structured through adverse outcome pathway mapping, provided mechanistic support for PHMG-P–induced chemical pneumonitis, collectively establishing a causal association between PHMG-P exposure and pneumonitis.

## INTRODUCTION

Pneumonia is commonly caused by infectious agents, such as bacteria, viruses, or fungi, leading to inflammation of the alveoli and terminal bronchioles. However, chemical pneumonitis—a non-infectious form of lung inflammation resulting from inhaled toxicants—shares overlapping clinical and radiologic features with infectious pneumonia. Throughout this study, the term chemical pneumonitis is used to denote toxicant-induced, non-infectious inflammatory lung injury, whereas pneumonia refers to the broader diagnostic categories used in epidemiological datasets (International Classification of Diseases 10th revision [ICD-10] J12-J18). Chemical pneumonitis arises when direct exposure to chemical substances triggers lung inflammation that often mimics the clinical and radiological presentation of infectious pneumonia. These patients do not respond to antimicrobial therapy and instead require immediate cessation of exposure or supportive treatment [1].

Humidifier disinfectant (HD) incidents in Korea were significant public health events involving widespread inhalation of toxic disinfectants, primarily polyhexamethylene guanidine phosphate (PHMG-P). Before official epidemiological investigations were initiated, the medical community reported a case series describing severe lung injury of unknown cause in the absence of clearly identified microbial or chemical etiologies [2]. Subsequent retrospective analyses and toxicological studies ultimately delineated these conditions as humidifier disinfectant-associated lung injury (HDLI) [3]. Importantly, many early cases presented with clinical symptoms and radiologic findings that closely resembled typical pneumonia (ICD-10 J12-J18), making accurate clinical differentiation challenging. Later analyses indicated that a substantial proportion of these cases were chemically rather than biologically induced. This growing body of evidence highlights the urgent need for a systematic reassessment of pneumonia diagnoses among individuals exposed to HDs.

Various toxic substances, both inorganic (chlorine, ammonia, nitrogen oxides, phosgene, cadmium, mercury) and organic (paraquat, nitrofurantoin), are known to cause chemical pneumonitis. Nitrogen dioxide (NO₂) gas is classically associated with “silo-filler’s disease,” a condition historically observed in agricultural settings. Inhaled NO₂ reacts with water in the airways to generate nitric acid, causing caustic injury to the airway and alveolar epithelium. Clinically, exposure to NO₂ manifests as acute chemical pneumonitis and bronchiolitis obliterans, typically presenting within hours with rapid onset of cough, pulmonary edema, and hypoxemia [4,5]. Similarly, chlorine gas—commonly encountered in industrial accidents or chemical warfare—induces immediate chemical burns of the airways and lung tissue. Upon contact with airway surface fluids, chlorine reacts to form hydrochloric acid and reactive oxidants, resulting in epithelial injury, acute pneumonitis with pulmonary edema, and increased risk of secondary reactive airways dysfunction syndrome [6].

Because of diagnostic ambiguity, biologically plausible mechanisms, and extensive public exposure following the HD incident, determining the causal association between PHMG-P and pneumonitis is crucial. This study aims to address this gap by integrating evidence from population-based epidemiological analyses, animal toxicology studies, and *in vitro* mechanistic studies. Utilizing the adverse outcome pathway (AOP) framework alongside structured evidence-grading criteria, we evaluate the certainty of the causal relationship and present scientific evidence supporting PHMG-P-induced chemical pneumonitis.

## MATERIALS AND METHODS

We used an integrative framework to evaluate the association between PHMG-P exposure and the development of chemical pneumonitis in this study. The evidence was assessed using a structured weight-of-evidence methodology to ensure scientific rigor and transparency [7]. This approach incorporates principles from the Office of Health Assessment and Translation (OHAT) [8], the AOP framework, and modified evidence grading criteria adapted from the Grading of Recommendations Assessment, Development and Evaluation system [9], as well as international chemical risk-assessment guidance [10,11].

Details of the evidence-integration framework used in the present study have been described previously [12,13]. Relevant studies were identified through a systematic literature search and were evaluated for methodological quality, internal validity, and relevance to human health. Epidemiological and toxicological lines of evidence were assessed separately using predefined criteria and were subsequently integrated to determine the overall confidence in a causal relationship. This triangulated assessment was guided by established best practices for combining diverse evidence streams in environmental health research [14,15].

### Identifying epidemiological evidence

We conducted a systematic review of epidemiological studies on HD exposure and respiratory diseases, focusing on pneumonia (ICD-10 J12-J18) and chemical pneumonitis (ICD-10 J68). J12 to J18 refer to pneumonia caused by viruses not otherwise classified, *Streptococcus pneumoniae, Haemophilus influenzae*, other specified or unspecified bacterial and infectious agents, pneumonia in diseases classified elsewhere, and pneumonia due to unspecified organisms. Thus, pneumonia usually refers to infectious lung inflammation, whereas pneumonitis is non-infectious and often chemically induced. During the Korean HD incident, many cases initially coded as pneumonia were later reclassified as chemical pneumonitis.

Following the population, intervention/exposure, comparator, outcome (PICO/PECO) framework and the Preferred Reporting Items for Systematic Reviews and Meta-Analyses (PRISMA) guidelines, we searched PubMed, Web of Science, and the Korea Environment Library using MeSH and free-text terms (e.g., “humidifier disinfectant,” “PHMG-P,” “PGH,” “pneumonia,” “chemical pneumonitis”). In addition to studies directly conducted by the Ministry of Environment, the search included domestic and international peer-reviewed articles, reports, and guidelines from government and research institutions. Eligible studies employed valid epidemiological designs, reported ICD-based outcomes, and defined HD exposure or specific disinfectant agents. Only a limited number of studies published in Korea were found. Statistical approaches included age-period-cohort (APC), difference-in-differences (DID), and interrupted time-series (ITS) analyses in the health-damage claimant cohort, with ITS providing ingredient-specific evidence for PHMG-P and oligo(2-(2-ethoxy)ethoxyethyl guanidinium chloride) (PGH).

Study quality and risk of bias were appraised using the OHAT Risk of Bias Tool, the Strengthening the Reporting of Observational Studies in Epidemiology (STROBE) checklist, and the Risk of Bias in Non-randomized Studies of Exposure (ROBINS-E) tool, with emphasis on exposure classification, temporality, outcome validity, and adequacy of comparison groups. Each study was assigned an initial confidence rating (high, moderate, low, unclassifiable), which was revised through upgrading (large effect, dose–response, consistency, minimal confounding) or downgrading (bias, indirect exposure, heterogeneity, imprecision, publication bias). The overall strength of evidence was categorized as “sufficient,” “suggestive,” “unclassifiable,” or “unrelated,” following criteria adapted from the United States Environmental Protection Agency OHAT framework. A summary table of ratings and key upgrade/downgrade factors (Supplementary Material 1) has been added to improve transparency.

### Identifying toxicological evidence

**Figure 1.**
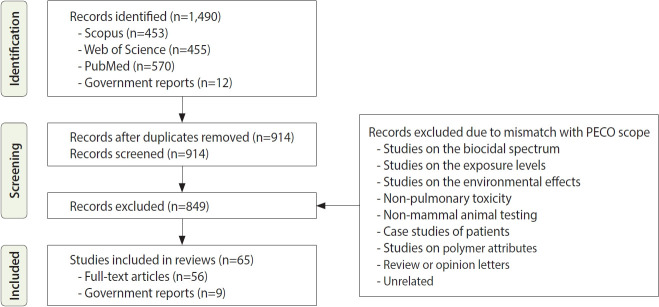
Preferred Reporting Items for Systematic Reviews and Meta-Analyses (PRISMA) flowchart for the systematic review of toxicological studies on polyhexamethylene guanidine phosphate–induced respiratory toxicity, focusing on chemical pneumonitis. PECO, population, exposure, comparator, outcome.

We systematically reviewed toxicological studies to assess the respiratory effects of PHMG-P exposure, with particular attention to chemical pneumonitis. Literature searches were conducted in PubMed, Web of Science, and Scopus up to March 2025 using the following search strings: (“polyhexamethylene guanidine” OR “PHMG-P” OR “humidifier disinfectant”). Studies were evaluated for quality according to the Toxicological Data Reliability Assessment Method [16], and only studies that met its validity criteria were included. The search strategy followed the PECO framework, targeting *in vivo* and *in vitro* studies investigating respiratory toxicity of PHMG-P. Inclusion criteria were: (1) original toxicological studies evaluating PHMG-P; (2) exposure via inhalation or intratracheal instillation; (3) outcomes describing histopathological lung lesions, bronchoalveolar lavage fluid (BALF) parameters, cytokine or oxidative stress markers, epithelial barrier injury, or immune dysregulation. Studies were excluded if they fell outside the predefined PECO scope, including studies focused on non-respiratory outcomes, non-mammalian models, biocidal efficacy or physicochemical properties without toxicological endpoints, clinical case reports or epidemiological studies without experimental data, and review or opinion articles. The study selection and screening process is summarized in Figure 1.

Evidence from these studies was synthesized into an AOP framework that links the molecular initiating event to cellular and tissue-level key events and ultimately to adverse outcomes, including irreversible structural damage and impaired lung function. Each study was evaluated for reliability, relevance, and consistency with human disease, and confidence in the AOP model was determined using Organization for Economic Cooperation and Development AOP Development and Evaluation Guidelines [10]. Overall, toxicological evidence demonstrated a biologically plausible and causally consistent pathway by which PHMG-P induces chemical pneumonitis.

### Integration of evidence and causal inference

Findings from the epidemiological and toxicological domains were triangulated to derive an integrated conclusion. A verified association was established when both evidence streams were rated as “sufficient” or “suggestive.” If only toxicological evidence was sufficient, but epidemiological findings were limited because of outcome rarity or study design constraints, an upgraded classification was considered, particularly when supported by consistent clinical observations.

Through this comprehensive and methodologically rigorous process, we aimed to establish a causal link between PHMG-P exposure and chemically induced pneumonitis and provide an evidence-based foundation for clinical, regulatory, and public health actions.

### Ethics statement

This study is a review article based on previously published literature and does not involve any new studies with human participants or animals. Therefore, approval from an institutional review board or an ethics committee was not required.

## RESULTS

### Epidemiological evidence linking humidifier disinfectant exposure to pneumonitis

Two epidemiological studies investigated the association between PHMG-P–containing HD exposure and pneumonitis. These studies comprised analyses of both the general population and health-damage claimants and employed diverse statistical designs, including APC, DID, and ITS analyses [17,18].

#### APC analysis

This national-level study demonstrated that children aged 5 years or less had an extremely high relative risk (RR) of pneumonia (RR, 61.27 for males and 62.02 for females) compared with individuals aged 14 years, who had the lowest incidence. Elevated risks were also observed among individuals born between 1938 and 1949 (RR, 14.57 for males and 15.48 for females) and between 1950 and 1969 (RR, 3.13 for males and 3.41 for females). The risk declined sharply after 2012, consistent with the market withdrawal of PHMG-P–based disinfectants (Table 1) [17].

#### DID analysis

By comparing pneumonia episodes during the pre-exposure period (2003-2012) and post-exposure period (2013-2019), it was estimated that 25% of cases could be attributed to HD exposure. Notably, the highest attributable fraction (51.4%) was observed in individuals aged 6-18 years. The total excess morbidity was 528,646 cases, including 222,200 cases in children aged 5 years or less and 86,474 cases in individuals born between 1990 and 1999 (Table 1) [17].

#### ITS analysis in the health damage claimant cohort

The risk of pneumonia increased shortly after the use of PHMG-P–containing HDs, peaked at 6 months, and remained elevated for up to 10 years (120 months). Excess rate ratios (ERRs) were estimated using Poisson regression models with person-months as the offset, comparing pneumonia episodes before and after PHMG-P/PGH exposure. Higher ERRs were observed in cases with high disease severity. ERR estimates were elevated at severity levels 5 (ERR, 17.94; 95% confidence interval [CI], 11.70 to 27.50), 4 (ERR, 18.28; 95% CI, 12.96 to 25.80), and 3 (ERR, 17.63; 95% CI, 13.10 to 23.70). Statistically significant ERRs were also detected at severity levels 1 and 2, supporting a severity-dependent effect of PHMG-P/PGH exposure on pneumonia episodes (Table 2) [18].

### Toxicological evidence supporting polyhexamethylene guanidine phosphate-induced chemical pneumonitis

We conducted a systematic review of relevant toxicological studies to assess PHMG-P–induced chemical pneumonitis. A total of 1,490 records were identified through database searches and government sources. After removal of duplicates and PECO-based screening, 65 studies (56 full-text articles and 9 government reports) met the eligibility criteria and were included in the final review (Figure 1). A detailed list of these studies is provided in Supplementary Material 2.

### *In vivo* animal studies

Multiple aerosol-exposure and intratracheal instillation studies in Sprague–Dawley (SD) rats and BALB/c mice consistently demonstrated that PHMG-P induced acute bronchial injury, characterized by epithelial damage and inflammation. Rats exposed to PHMG-P for 3 weeks (5 days per week) exhibited thickening of the alveolar walls and infiltration of inflammatory cells [19]. BALF analysis after PHMG-P exposure revealed marked neutrophilia and elevated protein concentrations, indicating compromised epithelial integrity [20]. Moreover, time-course studies showed that epithelial damage and inflammation persisted even after cessation of PHMG-P exposure, suggesting limited reversibility (Table 3) [21].

Long-term *in vivo* studies demonstrated that prolonged PHMG-P exposure led to persistent lung injury characterized by chronic inflammation and fibrosis. In a 13-week whole-body inhalation study, SD rats exposed to PHMG-P developed pronounced bronchiolar inflammation, epithelial hyperplasia, and interstitial fibrosis, with histopathological changes partially persisting after a 24-week recovery period [22]. These findings provided direct evidence of irreversible structural alterations consistent with chronic chemically induced pneumonitis. These chronic exposure models reflected the persistent nature of PHMG-P–induced lung injury observed in epidemiological studies and provided mechanistic insights into its progression toward irreversible respiratory dysfunction.

Molecular profiling supported these pathological observations. Transcriptomic analysis of lung tissues revealed persistent upregulation of pro-fibrotic and inflammatory genes, including TGF-β1, Col1A1, and MMP12, indicating activation of fibrogenic pathways [23]. Mechanistic analyses showed that PHMG-P induces epithelial–mesenchymal transition (EMT) and upregulates cytokines involved in tissue remodeling [23-25]. These events are accompanied by a cytokine milieu shift toward Th1 and Th17 profiles, characterized by increased production of interferon-gamma (IFN-γ) and interleukin (IL)-17A, thereby promoting sustained neutrophilic inflammation (Table 3) [26].

### *In vitro* mechanistic studies

PHMG-P exposure in lung epithelial cells resulted in increased reactive oxygen species (ROS) production, triggered mitochondrial depolarization, and caused DNA fragmentation [27]. In an *in vitro* air–liquid interface model, PHMG-P impaired epithelial barrier function by disrupting tight junctions, indicating compromised mucosal integrity (Table 4) [19].

At the mechanistic level, PHMG-P activates organelle-stress pathways, particularly endoplasmic reticulum (ER) stress responses, resulting in CHOP-mediated apoptotic signaling [28]. Additionally, PHMG-P exposure leads to the release of damage-associated molecular patterns (DAMPs), which initiate toll-like receptor 4 (TLR4)–mediated NF-κB signaling and drive the expression of pro-inflammatory cytokines (Table 4) [29,30]. PHMG-P disrupts epithelial homeostasis via ROS-associated membrane damage and cytotoxicity across diverse cell types, collectively driving inflammatory activation, barrier dysfunction, and profibrotic signaling (Table 4) [31].

### Adverse outcome pathway framework

**Figure 2.**
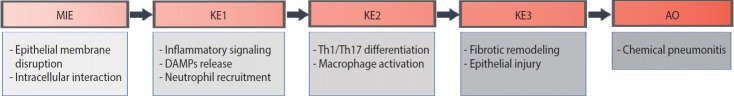
Conceptual diagram for the adverse outcome pathway of polyhexamethylene guanidine phosphate-induced chemical pneumonitis. MIE, molecular initiating event; KE, key event; AO, adverse outcome; DAMP, damage-associated molecular pattern; Th1, T-helper 1 cell; Th17, T-helper 17 cells.

The AOP framework was used to organize mechanistic evidence linking PHMG-P exposure to chemically induced pneumonitis (Figure 2). Inhalation of PHMG-P initiates a molecular initiating event through direct disruption of the respiratory epithelial barrier, resulting in cellular membrane damage, organelle dysfunction—particularly in the ER and mitochondria—and the release of DAMPs [31-34].

In key event (KE) 1, DAMPs activate pattern-recognition receptors, particularly TLRs, leading to NF-κB signaling and the release of pro-inflammatory cytokines such as IL-1β, IL-6, and TNF-α [19,30,32,35]. This inflammatory cascade promotes neutrophil and macrophage recruitment, consistent with *in vivo* studies demonstrating elevated neutrophils in BALF [20,25]. These events are pathologically consistent with early epithelial injury, luminal edema, and inflammatory cell infiltration [20]. KE 2 involves the adaptive immune response, including CD4+ T-cell differentiation into Th1 and Th17 subsets. Th1 cells release IFN-γ, sustaining macrophage activation, whereas Th17-derived IL-17A promotes neutrophilic infiltration and tissue injury [26]. This immune polarization, driven by sustained TLR signaling, results in chronic inflammation and impaired epithelial repair. In KE 3, prolonged immune activation initiates structural and fibrotic remodeling of the lung parenchyma. PHMG-P exposure induces alveolar collapse, tight-junction disruption, collagen deposition, and fibrosis, mediated by pathways such as TGF-β signaling and EMT [23,24]. Imaging studies in rat models have shown centrilobular nodules and diffuse alveolar damage, resembling radiological patterns observed in human HDLI [36].

The adverse outcome (AO) of chemical pneumonitis is characterized by sustained pulmonary inflammation, irreversible epithelial damage, impaired gas exchange, and chronic respiratory dysfunction. The proposed AOP is mechanistically coherent and aligns with established AOP-Wiki entries such as AOP 406 and AOP 320, which describe inflammation-associated respiratory outcomes following ACE2-mediated viral insults. These reference AOPs also emphasize TLR dysregulation, increased ROS production, and pro-inflammatory mediator release as central elements driving lung injury and hyperinflammation.

### Integration of evidence and causal inference

We conducted a structured integration of epidemiological and toxicological evidence to evaluate overall confidence in the causal relationship between PHMG-P exposure and chemically induced pneumonitis.

Epidemiological studies have reported statistically significant associations between HD use and increased pneumonia episodes, particularly among vulnerable subpopulations such as children and females of reproductive age. In addition, ERR analyses demonstrated a significant association with PHMG-P exposure.

Toxicological and mechanistic studies consistently show that PHMG-P induces airway epithelial injury, immune-cell infiltration, and fibrotic remodeling in both acute and chronic exposure models. *In vitro* assays identified molecular initiating events such as epithelial membrane disruption, oxidative stress, and inflammatory signaling via DAMP–TLR–NF-κB pathways, all converging on the AO of chemical pneumonitis.

Both lines of evidence were rated as “sufficient” in internal validity, coherence, and biological plausibility. When evaluated together, they reinforced each other and supported high-confidence causal inference. The observed convergence across epidemiological patterns, experimental pathology, and mechanistic plausibility substantiates PHMG-P as the causative agent of chemical pneumonitis.

## DISCUSSION

This study provides a comprehensive and integrative assessment of the causal relationship between PHMG-P exposure and chemically induced pneumonitis. By synthesizing epidemiological, toxicological, and mechanistic data within the AOP framework, we establish both the biological plausibility and the strength of evidence identifying PHMG-P as the primary causal agent underlying the HD incident in Korea.

### Epidemiological evidence strengthening causal inference

Multiple epidemiological analyses, including APC, DID, and ITS designs, consistently demonstrated a significant increase in the incidence of pneumonia and pneumonitis during periods of HD use. This increase was particularly evident among children, adolescents, and females of reproductive age. A markedly elevated RR (>60) was observed among children under 5 years of age in the APC analysis. Although full APC methods were previously detailed [17], in brief, we adjusted for potential confounders including age, period, cohort, income level, and residence. Results remained consistent across disease severities.

The epidemiological studies reported robust effect estimates based on national health database analyses and cohorts of HD-related health-damage claimants, with findings that aligned clearly and temporally with periods of HD exposure. In particular, the APC and DID analyses demonstrated excess morbidity attributable to HD exposure, estimating more than 220,000 excess pneumonia cases in children aged 5 years or less [17].

Importantly, these studies examined the entire population of HD users irrespective of the specific disinfectant component. To clarify the contribution of PHMG-P, ERR analyses were performed separately for individuals exclusively exposed to PHMG-P or PGH. These analyses showed a marked increase in pneumonia incidence following a dose–response gradient, with the most pronounced effects observed among severe cases (severity levels 4 and 5). This finding suggests a strong and specific association between PHMG-P/PGH exposure and chemically induced lung disease. As PGH users represented only a small fraction of the overall affected population, the majority of excess disease burden was attributable to PHMG-P exposure, reinforcing its likely role as the primary causative agent.

### Toxicological and mechanistic evidence supporting causality

A substantial body of toxicological and mechanistic research complements the epidemiological evidence linking PHMG-P exposure to chemically induced pneumonitis. Mechanistically, PHMG-P triggers both acute and chronic inflammatory processes. This response recruits neutrophils and macrophages to the site of injury, exacerbating inflammation and promoting tissue remodeling. These events ultimately drive irreversible structural alterations, including collagen deposition and alveolar fibrosis, consistent with chronic chemically induced lung injury. In addition to direct pulmonary toxicity, PHMG-P–induced barrier disruption likely facilitates secondary bacterial infections, particularly with S. pneumoniae, a major pathogen causing pneumonia. PHMG-P–mediated impairment of host defense, combined with pneumococcal virulence factors such as pneumolysin, creates a synergistic environment that amplifies respiratory system damage [37].

The AOP framework was used to integrate these mechanistic findings into a coherent biological narrative. This structured model delineates a biologically plausible sequence from the molecular initiating event—epithelial membrane disruption—to key events involving innate and adaptive immune activation, cytokine dysregulation, and fibrogenesis, culminating in the AO of chemically induced pneumonitis. The consistency of toxicological and mechanistic findings across independent studies, combined with their alignment with human clinical and epidemiological observations, provides strong biological plausibility for a causal relationship between PHMG-P exposure and chemically induced pneumonitis. The convergence of evidence from population-based analyses and experimental models substantially strengthens the inference of causality.

### Integration of evidence and public health implications

Pneumonia is usually caused by infectious pathogens. However, chemically induced pneumonitis—resulting from direct epithelial injury or immune dysregulation following toxic inhalation—can present with clinical and radiologic features similar to pneumonia, creating diagnostic uncertainty. This study integrates multidisciplinary evidence to establish a scientifically robust foundation supporting a causal relationship between PHMG-P exposure and chemically induced pneumonitis.

Beyond establishing causality, these findings have important implications for public health and regulatory policy. The widespread use of inhalable household disinfectants underscores the need to strengthen pre-market safety evaluations, particularly with respect to respiratory toxicity. The HD incidents in Korea exemplify the severe consequences of inadequate toxicological oversight and insufficient risk management regarding biocidal product use, which included delayed hazard recognition, misleading labeling, and regulatory blind spots. These shortcomings resulted in large-scale adverse health impacts.

Future chemical risk assessments should integrate mechanistic toxicology, experimental validation, and population-based epidemiology to prevent similar public health crises. Furthermore, systematic long-term health monitoring of individuals previously exposed to PHMG-P is essential to mitigate potential chronic respiratory complications. In summary, consistent high-quality evidence from multiple scientific disciplines provides a scientifically credible and policy-relevant foundation for concluding that exposure to PHMG-P is a specific cause of chemically induced pneumonitis. These findings reinforce the urgent need for precautionary regulatory actions and stringent chemical safety practices.

## CONCLUSION

Multidisciplinary evidence consistently supports PHMG-P as a causal agent of chemically induced pneumonitis. Integrated within the AOP framework, these findings demonstrate a biologically plausible sequence from molecular disruption to AOs and highlight the need for regulatory action and long-term health monitoring.

## Figures and Tables

**Figure f3-epih-47-e2025073:**
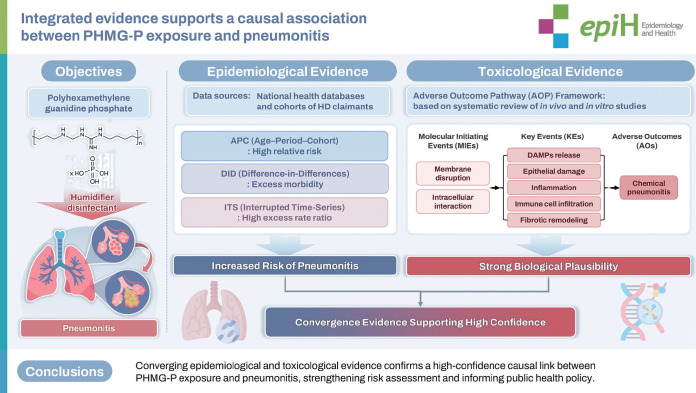


**Table 1.** Summary of epidemiological studies on pneumonia and HD exposure

Study design	Study population	Main findings	Effect size/risk
APC analyses [17]	National population	Highest risk in children aged ≤5 yr (RR> 60); high risk in 1938-1949 and 1950-1969 birth cohorts; risk decreased after 2012	Age effect, RR=12.84 and 13.00 (vs. 14 yr); cohort effect, RR=3.68 and 3.92 (vs. mean of all cohorts); period effect (vs. mean of all ages and cohorts) RR=0.89 and 0.90, in males and females, respectively
DID analyses [17]	National population	Compared periods exposed vs. non-exposed; highest attributable fraction in ages 6-18 and 1990-1999 birth cohorts, the highest excess episodes in children aged ≤5 yr and 2000-2012 birth cohorts	Attributable fraction: 25% overall, 51.4% in ages 6-18), and 48.9% in 1990-1999 birth cohort; Excess episodes 528,646-863,373 overall, 222,200 in ages ≤5, and 654,781 in the 2000-2012 birth cohort
ITS analyses [18], dynamic cohort	Health damage claims cohort	Immediate risk rise in post-PHMG-P exposure; persisted for up to 10 yr	Peaked at 6 mo; elevated risk for up to 120 mo; Risk of excess episodes after use compared to that before; 6-18 fold (see Table 2)

HD, humidifier disinfectant; APC, age-period-cohort; DID, difference-in-differences; ITS, interrupted time-series; RR, relative risk; PHMG-P, polyhexamethylene guanidine phosphate.

**Table 2.** Excess risk of pneumonia episodes 60 months before and after HD use in PHMG-P/PGH-only users

Severity level^1^	Excess episodes		After vs. Before
	Before	After	ERR (95% CI)^2^
1	268	2,682	6.48 (5.72, 7.34)
2	194	2,466	7.73 (6.68, 8.94)
3	44.81	1,300	17.63 (13.10, 23.70)
4	33.47	1,007	18.28 (12.96, 25.80)
5	21.85	645	17.94 (11.70, 27.50)

HD, humidifier disinfectant; PHMG-P, polyhexamethylene guanidine phosphate; PGH, oligo(2-[2-ethoxy]ethoxyethyl) guanidine hydrochloride; ERR, excess rate ratio; CI, confidence interval; ICD-10, International Classification of Diseases 10th revision.

1 Disease severity level defined by diagnostic classification and care setting in health insurance claim data: Level 1=ICD-10 J12-J18 codes listed in the primary, secondary, or other diagnosis categories for inpatient or outpatient care; Level 2=ICD-10 J12-J18 codes listed as primary or secondary diagnoses for inpatient or outpatient care; Level 3=ICD-10 J12-J18 codes listed as primary or secondary diagnoses for inpatient care with a hospital stay of ≥1 day; Level 4=ICD-10 J12-J18 codes listed as primary or secondary diagnoses for inpatient care with a hospital stay of ≥7 days; Level 5=ICD-10 J12-J18 codes listed as primary or secondary diagnoses for inpatient care with a hospital stay ≥14 days, or associated with death within a year, intensive care unit admission, mechanical ventilation, or cardiopulmonary resuscitation.

2 ERR=(excess rate after use)/(excess rate before use); After: during the 60-month period following each individual’s cessation of HD use; Before: during the 60-month period before each individual cessation of HD use; To calculate the ERRs, the denominator for PHMG-P/PGH users was 101,371 person-months for 60 months before use and 166,891 person-months for 60 months after use.

**Table 3.** Summary of animal studies evaluating PHMG-P-induced chemical pneumonitis

Title	Species	Exposure method	Key events	Ref (year)
PHMG-P aerosol particles induce pulmonary inflammatory and fibrotic responses	Rats (Sprague Dawley)	Nose-only (3 wk)	Epithelial damage, inflammation	[19] (2016)
Changes in expression of cytokines in polyhexamethylene guanidine-induced lung fibrosis in mice: Comparison of bleomycin-induced lung fibrosis	Mice (C57BL/6)	Intratracheal injection (single)	Inflammation, immune infiltration	[20] (2018)
Transcriptomic analysis of polyhexamethyleneguanidine-induced lung injury in mice after a long-term recovery	Mice (C57BL/6)	Intratracheal injection (repeat)	Inflammation, epithelial damage	[21] (2021)
Inhalation toxicity of PHMG-P in rats: A 4-wk inhalation exposure and 24-wk recovery period study	Rats (Sprague Dawley)	Whole-body (4 wk)	Inflammation, squamous metaplasia, emphysema, fibrosis	[22] (2023)
Time-course transcriptomic alterations reflect the pathophysiology of PHMG-P-induced lung injury in rats	Rats (Sprague Dawley)	Whole-body (4 wk)	Pro-fibrotic and inflammatory gene activation	[23] (2019)
Akt and Notch pathways mediate PHMG-P-induced epithelial-mesenchymal transition via ZEB2	Mice (C57BL/6)	Intratracheal injection (single)	Epithelial damage, EMT induction	[24] (2019)
Polyhexamethylene guanidine accelerates the macrophage foamy formation mediated pulmonary fibrosis	Mice (C57BL/6)	Whole-body (3 wk)	Immune infiltration, macrophage foamy formation	[25] (2024)
Causal relationship between humidifier disinfectant exposure and Th17-mediated airway inflammation and hyperresponsiveness	Mice (BALB/c)	Intratracheal injection (repeat)	Innate inflammation, T-cell differentiation	[26] (2021)

PHMG-P, polyhexamethylene guanidine phosphate; EMT, epithelial–mesenchymal transition; CT, computed tomography; RNA, ribonucleic acid; Th17, T-helper 17 cells; Ref, reference.

**Table 4.** Summary of *in vitro* studies evaluating PHMG-P-induced chemical pneumonitis

Title	Cell line	Key events	Ref (year)
PHMG-P aerosol particles induce pulmonary inflammatory and fibrotic responses	Calu-3, THP-1, HMC-1 (Co-culture)	ROS generation, cytokine expression (IL-6, TNF-α, and TGF-β)	[19] (2016)
PHMG-P-induced ROS-mediated DNA damage caused cell cycle arrest and apoptosis in lung epithelial cells	A549	ROS generation, DNA damage, apoptosis	[27] (2019)
PHMG-P induces apoptosis through ER stress in lung epithelial cells	A549	ER stress, apoptotic cell death	[28] (2021)
PHMG-P-induced upregulation of MUC5AC via activation of the TLR-p38 MAPK and JNK axis	Calu-3	TLR-MAPK (p38/JNK) activation	[29] (2019)
The role of NF-κB signaling pathway in PHMG-P induced inflammatory response in mouse macrophage RAW264.7 cells	RAW 264.7	NF-κB activation, cytokine expression (IL-6, TNF-α, and IL-1β)	[0] (2015)
PHMG-P induces cytotoxicity through disruption of membrane integrity	A549, MCR-5, THP-1	ROS generation, membrane disruption	[31] (2019)

PHMG-P, polyhexamethylene guanidine phosphate; NF-κB, nuclear factor kappa-light-chain-enhancer of activated B cells; IL, interleukin; TNF-α, tumor necrosis factor alpha; ROS, reactive oxygen species; TGF-β, transforming growth factor beta; DNA, deoxyribonucleic acid; TLR, toll-like receptor; MAPK, mitogen-activated protein kinase; JNK, c-Jun N-terminal kinase; MUC5AC, mucin 5AC; ER, endoplasmic reticulum; Ref, reference.

## Supplementary materials

Supplementary Material 1. Determination of downgrading or upgrading factors in assessment of the confidence level of the cumulative evidence

Supplementary Material 2. List of toxicological studies included in the systematic review
