# Supplementary material for: Integrated evidence supports a causal association between PHMG-P exposure and pneumonitis
Source: Epidemiol Health. 2025 Dec 14;47:e2025073. doi: 10.4178/epih.e2025073 (PMC12884041; doi:10.4178/epih.e2025073)
Supplement: Supplementary Material 2. — List of toxicological studies included in the systematic review [file epih-47-e2025073-Supplementary-2.docx]

**Supplementary Material 2. List of toxicological studies included in the systematic review**

| **Type** | **Title** | **Year** | **Journal** | **DOI** |
| --- | --- | --- | --- | --- |
| Research paper | Humidifier disinfectant–associated interstitial lung disease in an animal model induced by polyhexamethylene guanidine aerosol | 2014 | American Journal of Respiratory and Critical Care Medicine | 10.1164/rccm.201404-0710LE |
|  | Polyhexamethyleneguanidine phosphate induces severe lung inflammation, fibrosis, and thymic atrophy | 2014 | Food and Chemical Toxicology | 10.1016/j.fct.2014.04.027 |
|  | The role of NF-kappa B signaling pathway in polyhexamethylene guanidine phosphate induced inflammatory response in mouse macrophage RAW2647 cells | 2015 | Toxicology Letters | 10.1016/j.toxlet.2015.01.005 |
|  | Establishment of mouse model for pulmonary inflammation and fibrosis by intratracheal instillation of polyhexamethyleneguanidine phosphate | 2016 | Journal of Toxicologic Pathology | 10.1293/tox.2015-0067 |
|  | Polyhexamethylene guanidine phosphate aerosol particles induce pulmonary inflammatory and fibrotic responses | 2016 | Archives of Toxicology | 10.1007/s00204-015-1486-9 |
|  | Analysis of genomic responses in a rat lung model treated with a humidifier sterilizer containing polyhexamethyleneguanidine phosphate | 2017 | Toxicology Letters | 10.1016/j.toxlet.2016.11.005 |
|  | Low-dose cadmium exposure exacerbates polyhexamethylene guanidine-induced lung fibrosis in mice | 2018 | Journal of Toxicology and Environmental Health, Part A | 10.1080/15287394.2018.1451177 |
|  | Changes in expression of cytokines in polyhexamethylene guanidine induced lung fibrosis in mice: Comparison of bleomycin-induced lung fibrosis | 2018 | Toxicology | 10.1016/j.tox.2017.11.017 |
|  | Lung fibroblasts may play an important role in clearing apoptotic bodies of bronchial epithelial cells generated by exposure to PHMG-P-containing solution | 2018 | Toxicology Letters | 10.1016/j.toxlet.2018.01.003 |
|  | Oleanolic acid acetate attenuates polyhexamethylene guanidine phosphate-induced pulmonary inflammation and fibrosis in mice | 2018 | Respiratory Physiology & Neurobiology | 10.1016/j.resp.2018.03.001 |
|  | Polyhexamethylene guanidine phosphate induces IL-6 and TNF-alpha expression through JNK-dependent pathway in human lung epithelial cells | 2018 | The Journal of Toxicological Sciences | 10.2131/jts.43.485 |
|  | Protective Effects of Nintedanib against Polyhexamethylene Guanidine Phosphate-Induced Lung Fibrosis in Mice | 2018 | Molecules | 10.3390/molecules23081974 |
|  | Time course of polyhexamethyleneguanidine phosphate-induced lung inflammation and fibrosis in mice | 2018 | Toxicology and Applied Pharmacology | 10.1016/j.taap.2018.02.013 |
|  | MicroRNA regulatory networks reflective of polyhexamethylene guanidine phosphate-induced fibrosis in A549 human alveolar adenocarcinoma cells | 2018 | Toxicology Letters | 10.1016/j.toxlet.2018.01.010 |
|  | Akt and Notch pathways mediate polyhexamethylene guanidine phosphate-induced epithelial-mesenchymal transition via ZEB2 | 2019 | Pharmacology | 10.1016/j.taap.2019.114691 |
|  | Guanidine-based disinfectants, polyhexamethylene guanidine-phosphate (PHMG-P), polyhexamethylene biguanide (PHMB), and oligo(2-(2-ethoxy)ethoxyethyl guanidinium chloride (PGH) induced epithelial-mesenchymal transition in A549 alveolar epithelial cells | 2019 | Inhalation Toxicology | 10.1080/08958378.2019.1624896 |
|  | Metabolomic study on bleomycin and polyhexamethylene guanidine phosphate-induced pulmonary fibrosis mice models | 2019 | Metabolomics | 10.1007/s11306-019-1574-6 |
|  | Polyhexamethylene guanidine phosphate damages tight junctions and the F-Actin architecture by activating Calpain-1 via the P2RX7/Ca(2+) signaling pathway | 2019 | Cells | 10.3390/cells9010059 |
|  | Polyhexamethylene guanidine phosphate-induced ROS-mediated DNA damage caused cell cycle arrest and apoptosis in lung epithelial cells | 2019 | The Journal of Toxicological Sciences | 10.2131/jts.44.415 |
|  | Polyhexamethylene guanidine phosphate-induced upregulation of MUC5AC via activation of the TLR-p38 MAPK and JNK axis | 2019 | Chemico-Biological Interactions | 10.1016/j.cbi.2019.03.030 |
|  | Polyhexamethyleneguanidine phosphate induces cytotoxicity through disruption of membrane integrity | 2019 | Toxicology | 10.1016/j.tox.2019.01.001 |
|  | The anti-fibrotic effects of CG-745, an HDAC inhibitor, in bleomycin and PHMG-induced mouse models | 2019 | Molecules | 10.3390/molecules24152792 |
|  | Time-course transcriptomic alterations reflect the pathophysiology of polyhexamethylene guanidine phosphate-induced lung injury in rats | 2019 | Inhalation Toxicology | 10.1080/08958378.2019.1707912 |
|  | Aesculetin attenuates alveolar injury and fibrosis induced by close contact of alveolar epithelial cells with blood-derived macrophages via IL-8 signaling | 2020 | International Journal of Molecular Sciences | 10.3390/ijms21155518 |
|  | Integration of transcriptomics, proteomics and metabolomics identifies biomarkers for pulmonary injury by Poly hexamethylene guanidine phosphate (PHMG-p), a humidifier disinfectant, in rats | 2020 | Archives of Toxicology | 10.1007/s00204-020-02657-x |
|  | TGF beta/Smad mediated the polyhexamethyleneguanide areosol-induced irreversible pulmonary fibrosis in subchronic inhalation exposure | 2020 | Inhalation Toxicology | 10.1080/08958378.2020.1836091 |
|  | A humidifier disinfectant biocide, polyhexamethylene guanidine phosphate, inhalation exposure during pregnancy induced toxicities in rats | 2021 | Journal of Hazardous Materials | 10.1016/j.jhazmat.2020.124007 |
|  | Causal relationship between humidifier disinfectant exposure and Th17-mediated airway inflammation and hyperresponsiveness | 2021 | Toxicology | 10.1016/j.tox.2021.152739 |
|  | Evaluation of polyhexamethylene guanidine-induced lung injuries by chest CT, pathologic examination, and RNA sequencing in a rat model | 2021 | Scientific Reports | 10.1038/s41598-021-85662-z |
|  | Evaluation of the long-term effect of polyhexamethylene guanidine phosphate in a rat lung model using conventional chest computed tomography with histopathologic analysis | 2021 | PLOS ONE | 10.1371/journal.pone.0256756 |
|  | Exposure to cigarette smoke exacerbates polyhexamethylene guanidine-induced lung fibrosis in mice | 2021 | The Journal of Toxicological Sciences | 10.2131/jts.46.487 |
|  | The liver X receptor agonist T0901317 reduces the inflammation of alveolar epithelial cells induced by polyhexamethylene guanidine through inhibition of the NFκB signaling pathway | 2021 | Annals of Translational Medicine | 10.21037/atm-21-6501 |
|  | Physical analysis reveals distinct responses of human bronchial epithelial cells to guanidine and isothiazolinone biocides | 2021 | Toxicology and Applied Pharmacology | 10.1016/j.taap.2021.115589 |
|  | Polyhexamethylene guanidine aerosol triggers pulmonary fibrosis concomitant with elevated surface tension via inhibiting pulmonary surfactant | 2021 | Journal of Hazardous Materials | 10.1016/j.jhazmat.2021.126642 |
|  | Polyhexamethylene Guanidine Phosphate induces apoptosis through endoplasmic reticulum stress in lung epithelial cells | 2021 | International Journal of Molecular Sciences | 10.3390/ijms22031215 |
|  | MTF1 Is Essential for the Expression of MT1B, MT1F, MT1G, and MT1H Induced by PHMG, but Not CMIT, in the Human Pulmonary Alveolar Epithelial Cells | 2021 | Toxics | 10.3390/toxics9090203 |
|  | Assessment of acute and repeated pulmonary toxicities of oligo(2-(2-ethoxy)ethoxyethyl guanidium chloride in mice | 2021 | Toxicological Research | 10.1007/s43188-020-00058-x |
|  | Transcriptomic Analysis of Polyhexamethyleneguanidine-Induced Lung Injury in Mice after a Long-Term Recovery | 2021 | Toxics | 10.3390/toxics9100253 |
|  | Exposure to polyhexamethylene guanidine exacerbates bronchial hyperresponsiveness and lung inflammation in a mouse model of ovalbumin-induced asthma | 2021 | Allergy, Asthma & Immunology Research | 10.4168/aair.2021.13.4.655 |
|  | Analysis of lung cancer-related genetic changes in long-term and low-dose polyhexamethylene guanidine phosphate (PHMG-p) treated human pulmonary alveolar epithelial cells | 2022 | BMC Pharmacology and Toxicology | 10.1186/s40360-022-00559-5 |
|  | Anti-fibrotic effect of pycnogenol® in a polyhexamethylene guanidine-treated mouse model | 2022 | Respiratory Physiology & Neurobiology | 10.1016/j.resp.2021.103802 |
|  | Comparative toxicity of polyhexamethylene guanidine phosphate in three strains of rats | 2022 | Molecular & Cellular Toxicology | 10.1007/s13273-021-00169-y |
|  | Disruption of membrane integrity as a molecular initiating event determines the toxicity of Polyhexamethylene Guanidine Phosphate depending on the routes of exposure | 2022 | International Journal of Molecular Sciences | 10.3390/ijms23063289 |
|  | Polyhexamethylene guanidine phosphate increases stress granule formation in human 3D lung organoids under respiratory syncytial virus infection | 2022 | Ecotoxicology and Environmental Safety | 10.1016/j.ecoenv.2021.113094 |
|  | Polyhexamethylene guanidine phosphate-induced necrosis may be linked to pulmonary fibrosis | 2022 | Toxicology Letters | 10.1016/j.toxlet.2022.03.009 |
|  | Evaluating the comparative MT1B, MT1F, MT1G, and MT1H expression in human pulmonary alveolar epithelial cells treated with polyhexamethylene guanidine-phosphate, chloromethylisothiazolinone/methylisothiazolinone, oligo(2-(2-ethoxy)ethoxyethyl guanidinium chloride, benzalkonium chloride, and sodium dichloroisocyanurate | 2023 | Molecular & Cellular Toxicology | 10.1007/s13273-022-00311-4 |
|  | Fibrinogen on extracellular vesicles derived from polyhexamethylene guanidine phosphate-exposed mice induces inflammatory effects via integrin beta | 2023 | Ecotoxicology and Environmental Safety | 10.1016/j.ecoenv.2023.114600 |
|  | Gene expression related to lung cancer altered by PHMG-p treatment in PBTE cells | 2023 | Molecular & Cellular Toxicology | 10.1007/s13273-022-00319-w |
|  | NecroX Improves Polyhexamethylene Guanidine-induced Lung Injury by Regulating Mitochondrial Oxidative Stress and Endoplasmic Reticulum Stress | 2023 | American Journal of Respiratory Cell and Molecular Biology | 10.1165/rcmb.2021-0459OC |
|  | Inhalation toxicity of polyhexamethylene guanidine-phosphate in rats: A 4-week inhalation exposure and 24-week recovery period study | 2023 | Chemosphere | 10.1016/j.chemosphere.2022.137232 |
|  | Longitudinal long term follow up investigation on the carcinogenic impact of polyhexamethylene guanidine phosphate in rat models | 2024 | Scientific Reports | 10.1038/s41598-024-57605-x |
|  | Next generation risk assessment of biocides (PHMG-p and CMIT/MIT)-induced pulmonary fibrosis using adverse outcome pathway-based transcriptome analysis | 2024 | Journal of Hazardous Materials | 10.1016/j.jhazmat.2024.134986 |
|  | Polyhexamethylene guanidine accelerates the macrophage foamy formation mediated pulmonary fibrosis | 2024 | Ecotoxicology and Environmental Safety | 10.1016/j.ecoenv.2024.116084 |
|  | Nicotinamide adenine dinucleotide phosphate oxidase 2 deletion attenuates polyhexamethylene guanidine-induced lung injury in mice | 2024 | Heliyon | 10.1016/j.heliyon.2024.e25045 |
|  | Deciphering the toxicity of polyhexamethylene guanidine phosphate in  lung carcinogenesis: Mutational profiles and molecular mechanisms | 2024 | Chemosphere | 10.1016/j.chemosphere.2024.143785 |
|  | Polyhexamethylene guanidine phosphate induces pyroptosis via reactive oxygen species-regulated mitochondrial dysfunction in bronchial epithelial cells | 2024 | Toxicology | 10.1016/j.tox.2024.153827 |
| government report | Inhalation study for risk factor related to mysterious lung disease | 2011 | Government report |  |
|  | A safety study for management of the existing chemicals (I) | 2013 | Government report |  |
|  | A Study on Health Effects of Toxic Chemical Contained in Household Chemical Products (I) | 2014 | Government report |  |
|  | Toxicological Evaluation to Investigate Health Effects of Humidifier Disinfectants | 2018 | Government report |  |
|  | Investigation of the Mechanism of PHMG and PGH-Induced Lung Injury and Health Effects Beyond the Lung, Including Developmental Toxicity in Fetal Animal Models | 2019 | Government report |  |
|  | Development of toxicological indicator discovery technology to identify disease-specific (respiratory and non-respiratory diseases) causality by humidifier disinfectant ingredients | 2020 | Government report |  |
|  | Translated Summary (I) of PHMG-P Inhalation Toxicity and Related Animal Study Results | 2021 | Government report |  |
|  | Health Risk Evaluation of Humidifier Disinfectant Components Reflecting Inhalation-Based Exposure Patterns | 2021 | Government report |  |
|  | Studies on mechanisms for cytotoxicity  induced by humidifier disinfectants of  PHMG-p and CMIT/MIT (I) | 2022 | Government report |  |
